# King's College Hospital Pageant

**Published:** 1924-01

**Authors:** 


### KING'S COLLEGE HOSPITAL PAGEANT

King's College Hospital has arranged with the Crystal Palace to hold a pageant on the last three days of May next for the benefit of the hospital. The Pageant Master is Mr. Patrick Kirwan. The episodes include the last stand of Boadicea against the Romans at Peckham (61 A.D.); the Marriage Festivities of Gotha the Proud and Goda the daughter of Clape at Kennington (1042); the Foundation of Merton Priory (1117); the Rising of the Peasants of Surrey and Kent (1381); the Passing of the Pilgrims at St. Thomas a-Watering (1386); King Henry V.'s Return from Agincourt through Southwark (1415); King Henry's Widow Takes Sanctuary at Bermondsey (1423); Honor Oak Receives the Virgin Queen with May Revels (1570); Alleyne Gives a College to Dulwich (1619); With Mr. Pepys at Southwark Fair (1668); "The Club," including Dr. Johnson, David Garrick and Hogarth, Visits Vauxhall Gardens (1770).

